# *Mycobacterium tuberculosis* Requires Cholesterol Oxidase to Disrupt TLR2 Signalling in Human Macrophages

**DOI:** 10.1155/2019/2373791

**Published:** 2019-12-01

**Authors:** Izabela Szulc-Kielbik, Michal Kielbik, Patrycja Przygodzka, Anna Brzostek, Jaroslaw Dziadek, Magdalena Klink

**Affiliations:** Institute of Medical Biology, Polish Academy of Sciences, Lodz, Poland

## Abstract

This study tested the hypothesis that *Mycobacterium tuberculosis* (Mtb) uses a cholesterol oxidase enzyme (ChoD) to suppress a toll-like receptor type 2- (TLR2-) dependent signalling pathway to modulate macrophages' immune response. We investigated the impact of Mtb possessing or lacking ChoD as well as TBChoD recombinant protein obtained from Mtb on the expression and activation of two key intracellular proteins involved in TLR2 signalling in human macrophages. Finally, the involvement of TLR2-related signalling proteins in an inflammatory/immunosuppressive response of macrophages to Mtb was evaluated. We demonstrate that wild-type Mtb but not the ∆*choD* mutant decreased the cytosolic IRAK4 and TRAF6 protein levels while strongly enhancing *IRAK4* and *TRAF6* mRNA levels in macrophages. Our data show that the TLR2 present on the surface of macrophages are involved in disturbing the signalling pathway by wild-type Mtb. Moreover, recombinant TBChoD effectively decreased the cytosolic level of TRAF6 and lowered the phosphorylation of IRAK4, which strongly confirm an involvement of cholesterol oxidase in affecting the TLR2-related pathway by Mtb. Wild-type Mtb induced an immunosuppressive response of macrophages in an IRAK4- and TRAF6-dependent manner as measured by interleukin 10 production. In conclusion, ChoD is a virulence factor that enables Mtb to disturb the TLR2-related signalling pathway in macrophages and modulate their response.

## 1. Introduction

Alveolar-resident macrophages, next to dendritic cells and neutrophils, underlie the first line of immune defence against *Mycobacterium tuberculosis* (Mtb). Macrophages' main role is to recognize, ingest, and destroy the pathogen and to recruit other immune cells to the infected site. The recognition of Mtb antigens (*via* pathogen-associated molecular patterns (PAMP)) by specific pathogen recognition receptors (PRRs) is crucial to initiate the host immune response. An important class of evolutionary conserved PRRs involved in the host response to Mtb infection is the toll-like receptors (TLRs), especially the type 2, 4, and 9 TLRs. Notably, the literature suggests that TLR2 seems to be an important receptor for the recognition and/or uptake of Mtb as well as for the development of immune response. However, there are also opposite reports indicating that TLR2 is not critical for triggering macrophage effectors' mechanisms by Mtb. The currently known and well-described mycobacterial PAMPs that are TLR2 agonists include lipoarabinomannan (LAM), manosylated lipoarabinomannan (manLAM), 19 kDa lipoprotein, and phosphatidyl-myo-inositol mannoside (PIM) [1–7].

Mycobacterial PAMP recognition by TLR2 on macrophages results in MyD88 recruitment to the toll/interleukin-1 receptor (TIR) domain of TLR2, followed by the recruitment of the IL-1 receptor-associated kinases 1 and 4 (IRAK1 and 4). The next step is the phosphorylation of tumour necrosis factor (TNF) receptor-associated factor (TRAF) 6, which in turn leads to further phosphorylation of target signalling proteins [8, 9]. The major result of the TLR2 triggering with mycobacterial ligands is the production and secretion of various chemokines, cytokines, and bactericidal agents including interleukin- (IL-) 8, IL-1*β*, IL-6, IL-12, TNF-*α*, and nitric oxide or reactive oxygen species by the macrophage cell. This action promotes bacteria killing and/or induces the adaptive immune cell response [9–13]. Moreover, it is worth mentioning that TLR2 may be used by *M. tuberculosis* to interfere with the host immune response. Mtb inhibits the responses of human and murine macrophages to interferon-*γ* using TLR2 and the MyD88 protein to avoid being killed by these cells [14, 15].

Despite numerous investigations, knowledge on the virulence factors and survival strategies of Mtb remains insufficient. However, the most known and best described virulence factors include the cell wall components such as LAM, manLAM, 19 kDa lipoprotein, and PIM. Their main action inhibits phagosome maturation, but they also exhibit an inhibitory effect on transcriptional activation of interferon-*γ* inducible genes in macrophages [16–18]. However, a better understanding of the Mtb mechanism to evade the immune response remains an important research goal. One mechanism for the survival and persistence of tubercle bacilli in host cells is their ability to accumulate, degrade, and utilize cholesterol as a source of carbon and energy [19–22]. Cholesterol in particular is required for the phagocytosis of mycobacteria by macrophages since this pathogen enters phagocytes through cholesterol-rich membrane microdomains [20, 23]. Moreover, the enzymes participating in the cholesterol degradation are considered Mtb virulence factors [19, 22]. One enzyme, 3*β*-hydroxysteroid : oxygen oxidoreductase, commonly known as cholesterol oxidase (ChoD), is a flavoenzyme involved (although not essential) in the first step of cholesterol degradation [24–26]. This extracellular enzyme appears to be present in two forms: secreted and Mtb cell-surface associated [27–31].

Our group has demonstrated that cholesterol oxidase is an important Mtb component, allowing the bacteria to survive intracellularly. Cholesterol oxidase's role as a virulence factor significantly affects macrophage functional activity and not cholesterol degradation. Our previous study [32] demonstrates that Mtb uses ChoD to decrease the bactericidal response of human macrophages, resulting in intracellular survival. Moreover, our previous studies suggested that the TLR2-mediated signalling pathway is strongly involved in the response of macrophages to Mtb, but their mutual relation was not previously clarified. Therefore, in this paper, we showed the intracellular events related to TLR2 signalling that occurs in macrophages infected with wild-type Mtb and its mutant lacking a functional copy of the gene encoding for ChoD.

## 2. Materials and Methods

### 2.1. Cell Culture

The human monocyte-macrophage cell line THP-1 is derived from a male and was purchased from ATCC, Manassas, VA, USA. THP-1 cells were grown in culture medium containing RPMI 1640 medium (Thermo Fisher Scientific, Waltham, USA) supplemented with 1 mM sodium pyruvate, 10% FBS, 0.05 mM *β*-mercaptoethanol, and 100 U/ml penicillin and 100 *μ*g/ml streptomycin (P/S) at 37°C in a humidified 5% CO_2_ environment. Cells were passaged every 3 days.

### 2.2. Bacterial Growth Conditions

The bacterial strains described in this study were based on *M. tuberculosis* H37Rv (ATCC). The engineering of the Mtb strain deficient in the ChoD enzyme (Δ*choD*) and the complemented strain with an intact *choD* gene (Δ*choD-choD*) was described previously [33].

Mtb strains were grown in Middlebrook 7H9 supplemented with 10% OADC enrichment and 0.05% Tween 80 for up to 6 days until the strains reached an optical density value of 1 at 600 nm. Thereafter, the bacteria were divided into equal portions and stored at -85°C. One week later, a portion of each strain was thawed, clumps were disrupted by multiple passages through a 25-gauge needle, serial dilution of bacteria were prepared, and the number of bacteria was determined using the colony forming unit (CFU) method [34].

### 2.3. TBChoD Recombinant Protein Acquirement

The *choD* gene (Rv 3409c) from *M. tuberculosis* with a length of 578 aa was amplified using primers TBChoD sense 5′cgagatctATGAAGCCGGATTACGACGTCCTG and TBChoD reverse 5′cgtctagaGCCCGCGTTGCTGACCGG flanked by sequences recognized by BglII and XbaI restriction enzymes, cloned into a pJet1.2 PCR cloning system (Fermentas), sequenced, and finally cloned into pJam2 plasmid in frame with 6xHis tag. The resultant, recombinant plasmid was named TBChoDJam-6xHis. Following this, the TBChoDJam-6xHis plasmid was electroporated into the *M. smegmatis* mc^2^155 strain and obtained electrotransformants were checked by plasmid isolation and its restriction analysis.

For purification of the TBChoD protein, two independent *M. smegmatis* mc^2^155 strains carrying TBChoDJam-6xHis plasmids were grown in 7H9 medium supplemented with 10% OADC to OD_600_ 1. Next, 5 ml of culture was inoculated to fresh 7H9 OADC (1 l) and cultured up to OD_600_ 0.2 before induction with 0.4% of acetamide. The protein induction was carried out for 18 hours, and then cultures were centrifuged (20 minutes, 4500 rpm, 4°C). The pellets were suspended in 10 ml of binding buffer supplemented with protease inhibitors and sonicated for 20 seconds for 4 times with 5-minute breaks on ice using MP FastPrep. Subsequently, the cultures were centrifuged (30 minutes, 4500 rpm, 4°C) and supernatants were filtrated and transferred onto nickel columns. The protein purification procedure was carried out according to the manufacturer's protocol (Thermo Fisher Scientific). The analysis of *M. smegmatis* lysates overproducing of TBChoD protein is shown in the Supplementary Materials (Suppl. [Supplementary-material supplementary-material-1]).

### 2.4. Macrophage Preparation

THP-1 cells (monocytes) were suspended in fresh culture medium, distributed onto 6- or 24-well plates at 3 × 10^6^/ml or 1 × 10^6^/ml (or 1 × 10^5^/ml for the MTT assay), respectively, and differentiated using 20 ng/ml of phorbol 12-myristate 13-acetate (PMA) during 24 hours of culture (37°C, 5% CO_2_) to obtain macrophages as previously described [34]. After removing the culture media with PMA, fresh culture media was added, and cells were further infected and/or treated with stimuli.

### 2.5. Macrophage Infection or Stimulation

Prior to macrophage infection, Mtb strains were thawed, washed twice in RPMI 1640 medium, and suspended in culture medium without P/S. After clump disruption by multiple passages through a 25-gauge needle, serial dilutions of the mycobacterial strains were prepared in culture medium without P/S.

Before infection, some phagocytes were treated with 10 *μ*M of the IRAK1/4 inhibitor for 30 minutes or with 35 *μ*g/ml of a mouse anti-human TLR2 monoclonal antibody, azide-free or with isotype control, azide-free (Novus Biologicals, Littleton, USA) for 1 hour. The antibody concentration was previously titrated [34]. The treated and untreated macrophages were infected with the wild-type, Δ*choD*, and Δ*choD-choD* Mtb strains at the multiplicity of infection (MOI) equal to 10 for 2 hours. Next, the noningested Mtb were removed by intensive washing of the macrophage monolayer with Hanks' balanced salt solution. Fresh culture medium was added, and the infected macrophages were cultured for 24 hours and then used in the experiments. Some of the untreated macrophages were also infected with Mtb strains at an MOI of 10 for 0.5 to 4 hours. After infection, the noningested bacteria were washed, and the phagocytes were immediately used. Notably, the macrophages infected with the mycobacteria were always cultured in a medium without antibiotics.

Noninfected macrophages were also stimulated for 24 hours with PMA (100 ng/ml), lipoteichoic acid (LTA; 10 *μ*g/ml), or human recombinant interleukin 1*β* (hrIL-1*β*, 10 ng/ml) and were used to determine the cytosolic signalling protein levels.

### 2.6. Macrophage Treatment with TBChoD Recombinant Protein

First, noninfected macrophages were treated for 24 hours with either 10 *μ*g/ml of TBChoD, 10 *μ*g/ml of TBChoD with the addition of 100 U/ml of polymyxin B, or 10 *μ*g/ml of TBChoD formerly digested with 500 *μ*g/ml of proteinase K (2 hours in 50°C). Then, the supernatants were collected and the concentration of TNF-*α* was determined using an ELISA kit. Next, noninfected macrophages were cultured with TBChoD recombinant protein at concentrations ranging from 0.1 to 25 *μ*g/ml for 24 hours and the viability of phagocytes (MTT test) as well as the expression of TLR2 (flow cytometry) were determined. To estimate the influence of TBChoD recombinant protein on IRAK4, TRAF6, and p-IRAK4 protein levels, macrophages were treated or not with 10 *μ*g/ml of TBChoD for 1, 2, and 4 hours and then lysed.

### 2.7. MTT Assay

After 24 hours of incubation with a range of TBChoD recombinant protein concentrations, supernatants were discarded and 100 *μ*l of 3-(4,5-dimethylthiazol-2-yl)-2,5-diphenyltetrazolium bromide (MTT) solution (2 mg/ml) was added to each well. Then, cells were incubated for 3 hours (37°C; 5% CO_2_) for MTT uptake and reduction. Furthermore, the remaining MTT was gently removed, 2-propranolol (200 *μ*l) was added to each well, and the absorbance was measured with dual wavelengths of 595 nm and 630 nm using a Multiskan RC plate reader (Labsystems, Helsinki, Finland) equipped with Genesis Lite software. Obtained results were presented as optical density (OD) value.

### 2.8. Surface Expression of TLR2

Mtb-infected and noninfected macrophages, as well as phagocytes treated with TBChoD recombinant protein, were detached from plates and suspended in Dulbecco's phosphate-buffered saline (D-PBS) containing 1% FBS. To prevent nonspecific anti-TLR2 antibody binding, we blocked the crystallisable fragment receptors (FcRs) in D-PBS supplemented with 10% human AB serum for 15 minutes at room temperature. Next, cells were washed twice in D-PBS/1% FBS and stained with 10 *μ*g/ml of a PE-conjugated mouse anti-TLR2 monoclonal antibody (Novus Biologicals) or with a PE-conjugated mouse IgG2a isotype control (Becton Dickinson, Franklin Lakes, USA) for 30 minutes at 4°C, or the cells remained untreated. Next, all of the samples were washed twice in D-PBS/1% FBS and then examined with a FACS LSR II BD flow cytometer (Becton Dickinson) equipped with BD FACSDiva Software. The results were analysed by WinMDI software, and the data are presented as the median fluorescence intensity (MFI), which correlates with the surface expression of the target molecule.

### 2.9. THP-1 Nucleofection and TRAF6 Silencing with siRNA

THP-1 cells were grown at a density of 4–5 × 10^6^/ml. TRAF6-siRNA (ON-TARGETplus Human TRAF6 (7189) siRNA SMARTpool) and nontargeting NT-siRNA (ON-TARGETplus Nontargeting Pool) (Dharmacon, Lafayette, USA) were delivered to THP-1 cells using an Amaxa® 4D-Nucleofector® X Unit and a SG Cell Line 4D-Nucleofector X Kit L (Lonza, Basel, Switzerland) according to the manufacturer's instructions (60–100 pmol/2.14 × 10^6^ cells). Nucleofected cells were seeded at 1 × 10^6^ cells/well (for infection and for immunoblot-ECL analysis) or 1.4 × 10^5^ cells/well (for RNA analysis) on a 24-well Advanced TC Plate. Fresh medium (1 ml) was added 4 hours post nucleofection, and differentiation started with 20 ng/ml PMA (37°C, 5% CO_2_). After 24 hours, total RNA was isolated for real-time analysis, and the medium was changed in the cells to prepare for the infection. Fresh culture medium with 10 ng/ml PMA was added prior to 24-hour incubation (37°C, 5% CO_2_). Next, the cells were infected with the Mtb strains as described above or an immunoblot-ECL analysis was performed. After 24 hours of culturing the infected macrophages, the supernatants were collected and stored at -80°C, and total RNA was isolated from cells as described below.

### 2.10. Immunoblot-ECL Analysis

Mtb-infected, noninfected, and stimulated macrophages as well as phagocytes treated with TBChoD recombinant protein were lysed for 30 minutes on ice using a lysing buffer (20 mM Tris, 150 mM NaCl, 1 mM EDTA, 1 mM EGTA, 1% Triton-X 100, 1 mM PMSF, and 1% Halt Protease and Phosphatase Inhibitor Cocktail). The amount of protein in each lysate was measured with a DC Protein Assay Kit (Bio-Rad, Hercules, USA). All samples were heated for 10 minutes at 100°C with 2× sample buffer (125 mmol/l Tris, 4% SDS, 20% glycerol, 2% *β*-mercaptoethanol, and bromophenol blue), and then equal amounts of the protein were resolved on a 10% mini-PROTEAN Precast TGX SDS-PAGE Gel (Bio-Rad). After electrophoresis, proteins were transferred from the gel and onto a PVDF membrane using the Trans-Blot Turbo Transfer System (Bio-Rad). The membranes were blocked in SuperBlock Blocking Buffer and then probed with the following four specific primary antibodies: (1) rabbit anti-IRAK4 polyclonal antibody (1 : 1000), (2) rabbit anti-phospho-IRAK4 (Thr345/Ser346) monoclonal antibody (1 : 1000), (3) rabbit anti-beta-actin monoclonal antibody (1 : 4000) (Cell Signaling Technology, Danvers, USA), or (4) rabbit anti-TRAF6 polyclonal antibody (1 : 1000) (Proteintech Group, Rosemont, USA). Next, the membranes were washed in 2× TBS-Tween 20, probed with horseradish peroxidase- (HRP-) conjugated goat polyclonal anti-rabbit IgG secondary antibody, and washed again in 2× TBS-Tween 20. Finally, the proteins were visualized using the Pierce ECL Western Blotting Substrate kit (Thermo Fisher Scientific), and the signals were detected on XAR film. Densitometric analysis of the blots was performed using the FluoroChem MultiImage FC Cabinet (Alpha Innotech Corporation, San Leandro, USA) and AlphaEaseFC Software 3.1.2. The results are presented as the optical density intensity (ODI) of the area under each band's peak.

### 2.11. Cytokine Assays

After 24 hours of incubation (37°C; 5% CO_2_), the supernatants from the noninfected and Mtb-infected macrophages were collected and stored at -80°C. The secreted cytokines were measured *via* BD Cytometric Bead Array Human Inflammatory Cytokines Kit. The sensitivity of the kit for the tested cytokines was as follows: IL-8—3.6 pg/ml; IL-1*β*—7.2 pg/ml; IL-6—2.5 pg/ml; IL-10—3.3 pg/ml; and TNF-*α*—3.7 pg/ml.

The level of TNF-*α* in supernatants collected from macrophages treated with ChoD recombinant protein was estimated using a Human TNF alpha ELISA Ready-SET-Go! Kit (Thermo Fisher Scientific) with a sensitivity of 4 pg/ml.

### 2.12. RNA Extraction and qRT-PCR Analysis

Total RNA was isolated from noninfected and Mtb-infected cells using the TRIzol Reagent (Thermo Fisher Scientific) according to the manufacturer's protocol. Next, the quantity and quality control of the isolated RNA was assessed using NanoDrop with ND 1000 Software (Thermo Fisher Scientific) and the 2100 Bioanalyzer (Agilent Scientific, Santa Clara, USA). The RNA template (2 *μ*g) was processed directly to cDNA synthesis using the Maxima First Strand cDNA Synthesis Kit for RT-qPCR (Thermo Fisher Scientific) following the manufacturer's instructions.

The human *β*-actin, *GAPDH*, *TRAF6*, *TLR2*, *IRAK4*, *IL-8*, and *IL-10* expression levels were quantified using a TaqMan Gene Expression Assay (Thermo Fisher Scientific) by real-time PCR using an ABI 7900HT Fast Real-time PCR system (Thermo Fisher Scientific) according to the manufacturer's protocol. Briefly, equal amounts of cDNA were amplified in triplicate for all TaqMan Assays in MicroAmp 96-well plates. Each sample was supplemented with 1 *μ*l of 20× TaqMan Assay, made up to 20 *μ*l using TaqMan 2× Universal PCR Master Mix (with AmpErase UNG) and processed by the following four steps: (1) 50°C/2 min, (2) 95°C/10 min, (3) 95°C/15 s, and (4) 60°C/60 s for up to 50 cycles. Each assay included blank controls with no template cDNA. The signal was collected at the end of every cycle point. The relative gene expression quantitation was calculated using the comparative CT (ΔΔCT) method [35]. The data were analysed with the ABI 7900HT RQ Manager Software v1.2 and DataAssist Software v3.01. The results are presented as the fold change in the gene expression normalized to the reference genes (*β*-actin and *GAPDH*) and relative to the control (noninfected macrophages).

### 2.13. Statistical Analysis

All data are presented as the means ± SEMs (Standard Error of the Mean) and were analysed with a nonparametric Mann-Whitney *U* test or a Wilcoxon's signed rank test using Statistica 13.0 software for Windows. RT-PCR data were analysed with *t*-tests using DataAssist Software v3.01 (Thermo Fisher Scientific). Statistical significance was defined as *p* ≤ 0.05.

## 3. Results

### 3.1. TLR2 Expression in Macrophages Infected with Mtb Strains

**Figure 1:**
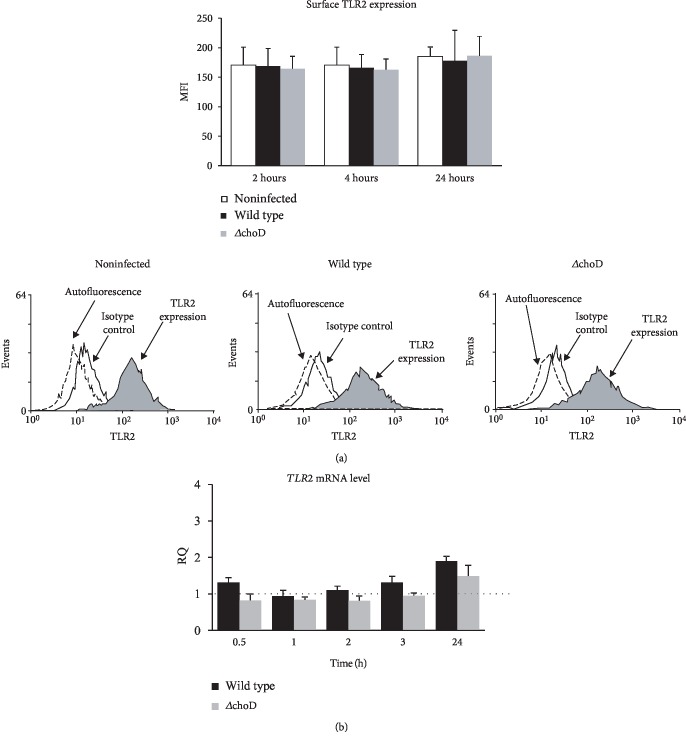
TLR2 surface expression and *TLR2* gene expression in Mtb-infected macrophages. The macrophages were infected with wild-type or Δ*choD* Mtb strains for 0.5 to 24 hours. (a) The cells were then stained with the PE-conjugated anti-TLR2 mAb or a specific isotype control for 30 minutes. The TLR2 expression on the macrophages was analysed using flow cytometry. The graphs show the mean values of the mean fluorescence intensity (MFI) ± SEM from 5 independent experiments. The representative histograms show the receptor expression level on the macrophages for both noninfected and infected macrophages. (b) The *TLR2* mRNA level was measured using qRT-PCR. The data are presented as the mean relative quantification (RQ) ± SEM from 3 independent experiments. The solid line marks the level of gene expression in control cells (noninfected macrophages). RQ represents the fold change in the gene expression in the infected macrophages compared to the noninfected macrophages, calculated using the ABI 7900HT RQ Manager Software v1.2 and DataAssist Software v3.01 (Thermo Fisher Scientific).

The TLR2 surface expression level determined by flow cytometry and presented as MFI values was similar between the noninfected and Mtb-infected macrophages, which was independent of the infection time (2, 4, or 24 hours) (Figure 1(a)). Moreover, we did not observe significant differences in the TLR2 expression on macrophages infected with wild-type or Δ*choD* Mtb. However, when measuring the *TLR2* gene expression, the mRNA level was lower in the Δ*choD*-infected macrophages than in wild-type-infected phagocytes; however, these differences were not statistically significant (Figure 1(b)).

### 3.2. IRAK4 and TRAF6 mRNA and Protein Expression in Mtb-Infected Macrophages

**Figure 2:**
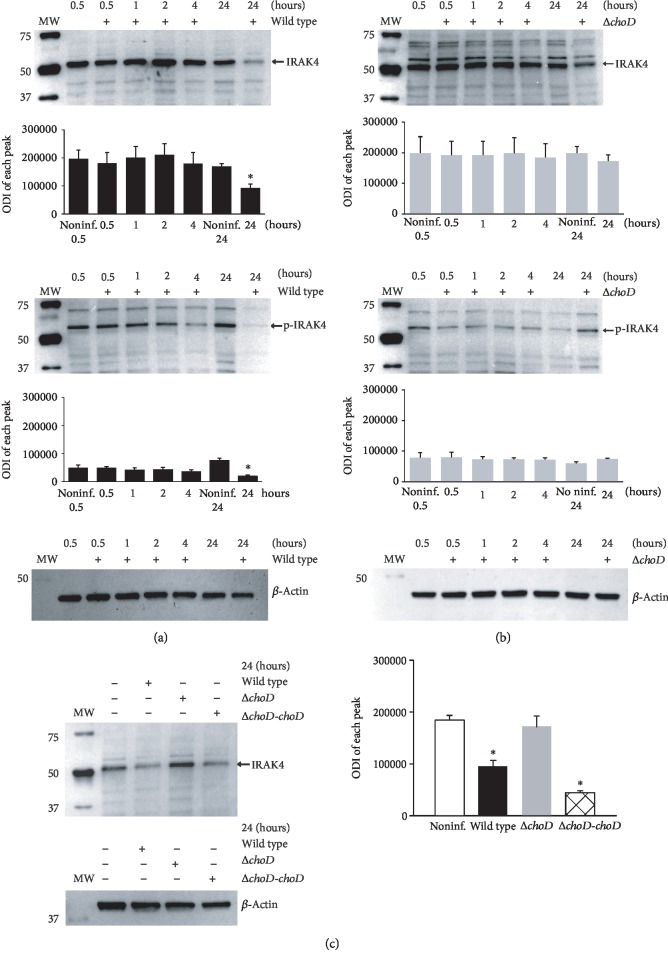
IRAK4 and p-IRAK4 protein levels in Mtb-infected macrophages. The macrophages were infected with (a) wild-type, (b) Δ*choD*, or (c) Δ*choD-choD* strains for 0.5 to 24 hours. The IRAK4 protein level was assessed using the immunoblot-ECL method. Representative immunoblots of the total and phosphorylated IRAK4 protein level are shown. The bands were quantified by densitometric analysis. The data are presented as the optical density intensity of the area under each band′s peak (ODI) ± SEM from 5 independent experiments. ^∗^Macrophages infected with the wild-type or Δ*choD-choD* vs. noninfected macrophages, *p* ≤ 0.02 (Wilcoxon's signed rank test).

IRAK4 is an important protein that initiates TLR2-mediated signalling events in response to Mtb infection [3, 36]. The total and phosphorylated IRAK4 protein level was assessed by immunoblot-ECL analysis. Our studies demonstrate that a short Mtb infection (0.5, 1, 2, and 4 hours) does not affect the total or phosphorylated IRAK4 protein level in wild-type (Figure 2(a)) or Δ*choD* mutant (Figure 2(b)) Mtb. Even though a slight decrease in p-IRAK4 was observed in macrophages infected for 4 hours with the wild-type Mtb, this decrease was not statistically significant (Figure 2(a)). *M. tuberculosis* displays a very long infection cycle inside human macrophages; hence, visible changes were observed 24 hours post infection. Although the mutant strain lacking the *choD* gene still had no impact on the total or phosphorylated IRAK4 protein level (Figure 2(b)), wild-type Mtb significantly decreased the total and phosphorylated IRAK4 protein level in the macrophages compared to the noninfected phagocytes (Figure 2(a)). The complemented Mtb strain induced a similar effect on IRAK4 as the wild-type (Figure 2(c)).

**Figure 3:**
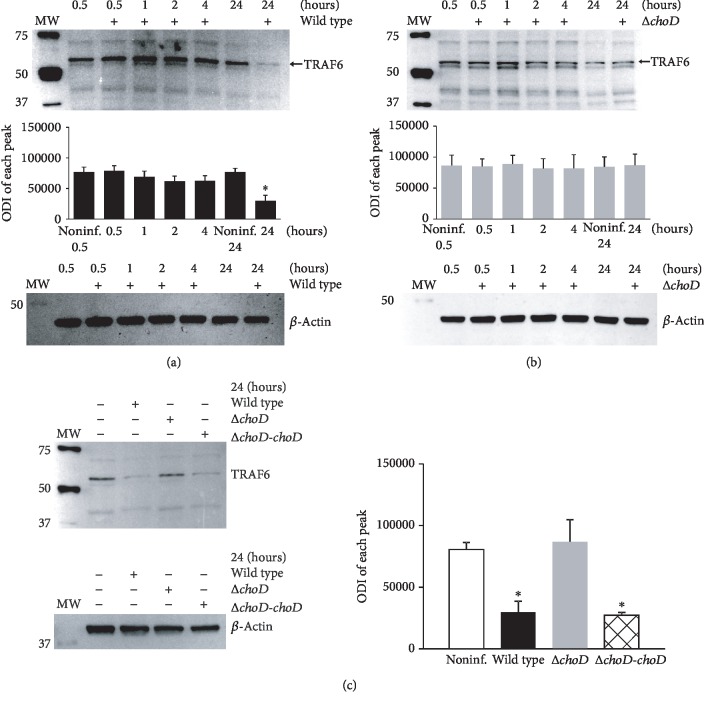
TRAF6 protein level in Mtb-infected macrophages. The macrophages were infected with (a) wild-type, (b) Δ*choD*, or (c) Δ*choD-choD* strains for 0.5 to 24 hours. The TRAF6 protein level was assessed using the immunoblot-ECL method. Representative immunoblots of the TRAF6 protein level are shown. The bands were quantified by densitometric analysis. The data are presented as the optical density intensity of the area under each band′s peak (ODI) ± SEM from 5 independent experiments. ^∗^Macrophages infected with the wild-type or Δ*choD-choD* vs. noninfected macrophages, *p* ≤ 0.04 (Wilcoxon's signed rank test).

Another protein involved in the TLR2-mediated signalling pathway is TRAF6, a downstream target for activated IRAK4 [3, 36]. We observed no significant effect of either Mtb strain on the TRAF6 level after a short infection. Similar to IRAK4, the notable changes in TRAF6 were observed 24 hours after infection. Notably, compared with noninfected macrophages, wild-type Mtb (Figure 3(a)) and the complemented strain (Figure 3(c)) but not Δ*choD* Mtb (Figure 3(b)) significantly decreased the TRAF6 protein level. These results indicate that Mtb needs cholesterol oxidase to decrease the TLR2-related signalling pathway activity. We would like to underline that the observed difference in the level of IRAK4 and TRAF6 in macrophages infected for 24 hours with wild-type Mtb or mutant strain is not related to a potential various bacteria load. As we described previously [32], macrophages ingested bacteria of both strains on a similar level. Moreover, the growth of Mtb strains (MOI = 1) inside phagocytes was similar until the 4th day post infection (CFU/ml for the wild-type amounted to 12,788 ± 3326; CFU/ml for the ∆*choD* mutant amounted to 15,686 ± 3557). The growth of the mutant strain was significantly impaired only on the 6th day post infection (CFU/ml for wild-type and ∆*choD* mutant amounted to 39,800 ± 7594 and 18,350 ± 7840, respectively). In this paper, in a control experiment using propidium iodide, we also determined the viability of the macrophages infected for 24 hours with Mtb strains (MOI = 10). The percentage of viable macrophages infected with wild-type strain or ∆*choD* mutant amounted to 85 ± 10 and 82 ± 11, respectively.

Additionally, to verify whether the observed cytosolic decrease in the IRAK4 protein is related to the fully active (alive) Mtb, we stimulated macrophages with a heat-killed wild-type strain (20 minutes, 80°C) and then assessed the level of protein. The results showed no difference in the IRAK4 protein level between the nonstimulated or heat-killed wild-type-stimulated phagocytes (data not shown).

**Figure 4:**
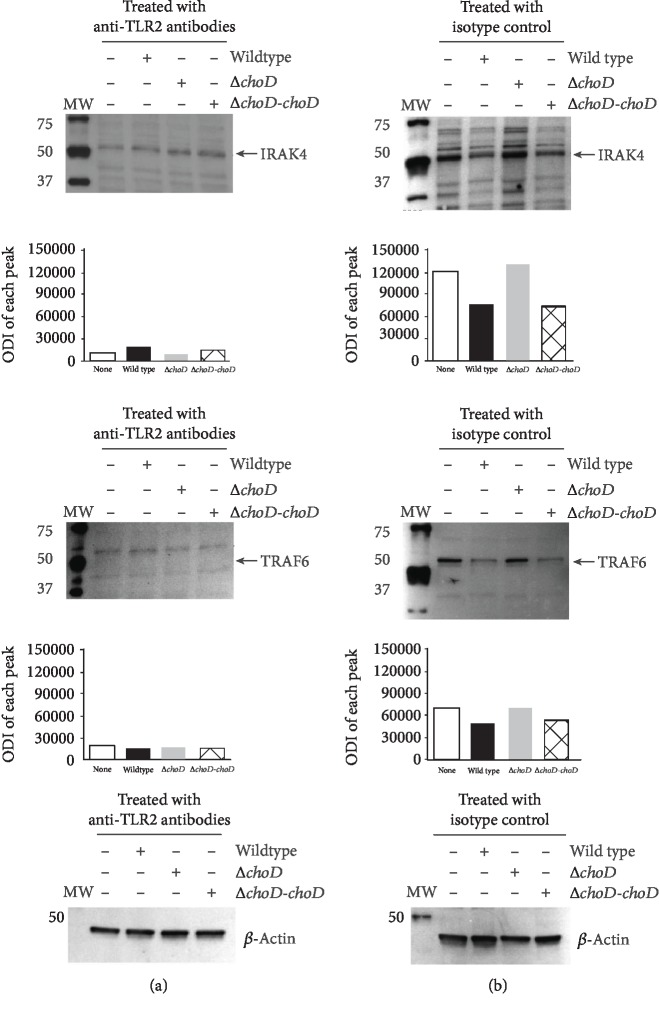
IRAK4 and TRAF6 protein levels in Mtb-infected macrophages with blocked TLR2 expression. The macrophages were incubated with an anti-human TLR2 monoclonal antibody (a) or isotype control (b) and then infected with the wild-type, Δ*choD*, or Δ*choD-choD* strains for 24 hours. Next, the cells were lysed, and the protein level was assessed using the immunoblot-ECL method. Representative immunoblots of the IRAK4 and TRAF6 protein levels are shown. The bands were quantified by densitometric analysis. The data are presented as the optical density intensity of the area under each band′s peak (ODI) ± SEM from 3 independent experiments.

To verify whether the observed loss in the protein level was dependent on the presence of the surface TLR2 during the phagocytosis process, we used monoclonal anti-TLR2 antibodies prior to the infection to block the TLR2 on the macrophage surface. As shown in Figure 4(a), the total IRAK4 and TRAF6 protein levels were the same for noninfected phagocytes and those infected with the wild-type, Δ*choD*, or complemented strain (Δ*choD-choD*) when TLR2 was blocked. It clearly indicates that the surface expression of TLR2 is required to initiate by wild-type Mtb the negative signal in macrophages that resulted in a lack of cytosolic IRAK4 and TRAF6 proteins. The level of both proteins in macrophages, treated with the isotype control and infected with Mtb strains, was the same as that observed when the surface TLR2 were not blocked with anti-TLR2 antibodies (Figure 4(b)).

**Figure 5:**
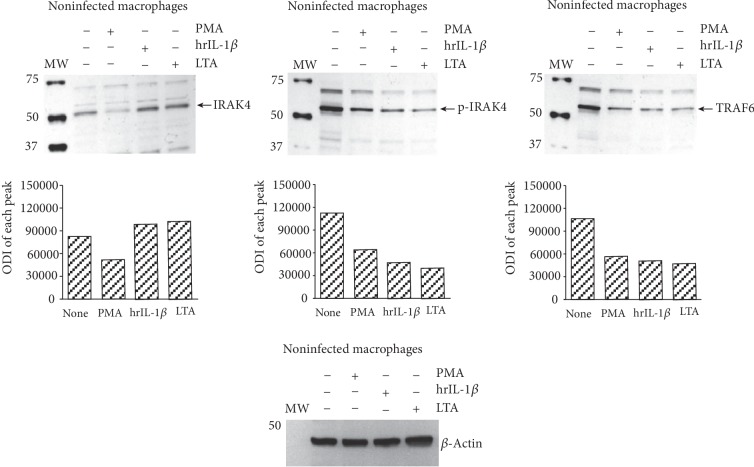
IRAK4 and TRAF6 protein levels in macrophages treated with PMA, IL-1*β*, or LTA. The macrophages were cultured for 24 hours in the presence of PMA, lipoteichoic acid (LTA), or human recombinant interleukin 1*β* (hrIL-1*β*). Next, the cells were lysed, and the IRAK4, p-IRAK4, and TRAF6 protein levels were assessed using the immunoblot-ECL method. Representative immunoblots of the IRAK4 and TRAF6 protein levels are shown. The bands were quantified by densitometric analysis. The data are presented as the optical density intensity of the area under each band's peak (ODI)±SEM from 2 independent experiments.

Previous studies demonstrate that prolonged TLR ligand stimulation leads to a decreased level of their signalling proteins [37, 38]. Thus, we investigated whether a 24-hour macrophage stimulation with LTA or hrIL-1*β* would influence the TLR2-mediated signalling pathway. Additionally, we activated cells with PMA. We observed a significant reduction in the IRAK4, p-IRAK4, and TRAF6 protein levels caused by the PMA action. Although incubating the macrophages with hrIL-1*β* or LTA did not alter the total IRAK4 protein level, the total TRAF6 and p-IRAK4 protein levels were notably reduced (Figure 5).

**Figure 6:**
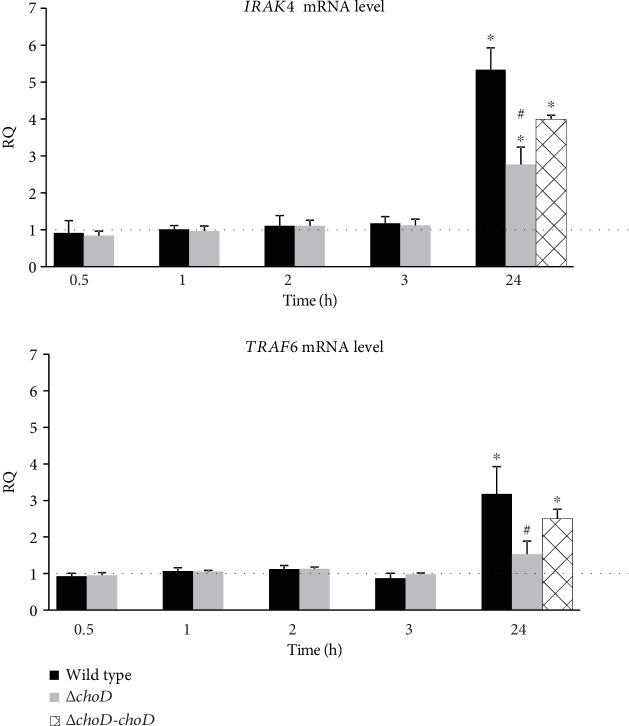
The *IRAK4* and *TRAF6* mRNA levels in Mtb-infected macrophages. The macrophages were infected with the wild-type, Δ*choD*, or Δ*choD-choD* strains for 0.5 to 24 hours, and the total RNA was isolated. The *IRAK4* and *TRAF6* mRNA level was measured using qRT-PCR. The data are presented as the mean relative quantification (RQ) ± SEM from 5 independent experiments. The solid line marks the level of gene expression in control cells (noninfected macrophages). RQ represents the fold change in the gene expression in the infected macrophages compared to the noninfected macrophages, calculated using the ABI 7900HT RQ Manager Software v1.2 and DataAssist Software v3.01 (Thermo Fisher Scientific). ^∗^Macrophages infected with wild-type, Δ*choD*, or Δ*choD-choD* Mtb vs. noninfected macrophages, *p* ≤ 0.03; ^#^Macrophages infected with Δ*choD* Mtb vs. macrophages infected with wild-type Mtb, *p* ≤ 0.04 (*t*-test).

The mRNA expression of the *IRAK4* and *TRAF6* genes were evaluated with TaqMan Gene Expression Assays using a real-time polymerase chain reaction. The results show that during a short time of phagocytosis, both the wild-type and Δ*choD* Mtb caused no change in the *IRAK4* and *TRAF6* mRNA levels compared to the noninfected macrophages (Figure 6). However, extending the incubation time to 24 hours significantly increased the phagocyte gene expression. Moreover, the increase in the *IRAK4* and *TRAF6* mRNA levels was statistically significant in the wild-type-infected macrophages compared with that in the Δ*choD*-infected macrophages (Figure 6). Phagocyte infection with a complemented strain (Δ*choD-choD*) affected the gene expression in a similar manner as the wild-type Mtb.

### 3.3. The Effect of TBChoD Recombinant Protein on TLR2 Pathway in Macrophages

To confirm the importance of ChoD as a virulent factor of Mtb, we cloned *choD* into pJam2 plasmid in frame with 6xHis tag (see Section 2.3 of Materials and Methods), introduced it into the *M. smegmatis* mc^2^155 strain, purified TbChoD, and finally used it for the treatment of macrophages.

In the initial experiment, we analysed whether the obtained TBChoD protein had any influence on the viability of macrophages. With an MTT test, we verified that the TBChoD protein used at a high range of concentrations (0.1-25 *μ*g) did not induce macrophages' death during 24 hours of treatment (OD value for nontreated macrophages was 0.785 ± 0.02, while OD value for macrophages treated with 25 *μ*g of TBChoD protein was 0.782 ± 0.05). Next, we tested if any possible effect of TBChoD on macrophages would be caused by protein contamination with LPS or lipid components derived from Mtb cell walls. The addition of polymyxin B did not change the ability of TBChoD (10 *μ*g/ml) to induce TNF-*α* release by macrophages (214 ± 27 pg/ml for TBChoD, 226 ± 44 pg/ml for TBChoD and polymyxin B, and 23 ± 6 pg/ml for control cells). Moreover, LPS (10 ng/ml) stimulated macrophages to secrete TNF-*α* at the level of 1091 ± 167 pg/ml. In contrast, proteinase K inhibited TNF-*α* secretion from 214 ± 27 pg/ml for TBChoD to 43 ± 3 pg/ml for TBChoD and proteinase K. What is more, this protein (10 *μ*g/ml) was active in the stimulation of macrophages to release various cytokines: IL-6 amounted to 24.9 pg/ml, IL-10 amounted to 10.3 pg/ml, and IL-8 amounted to 11,854 pg/ml.

**Figure 7:**
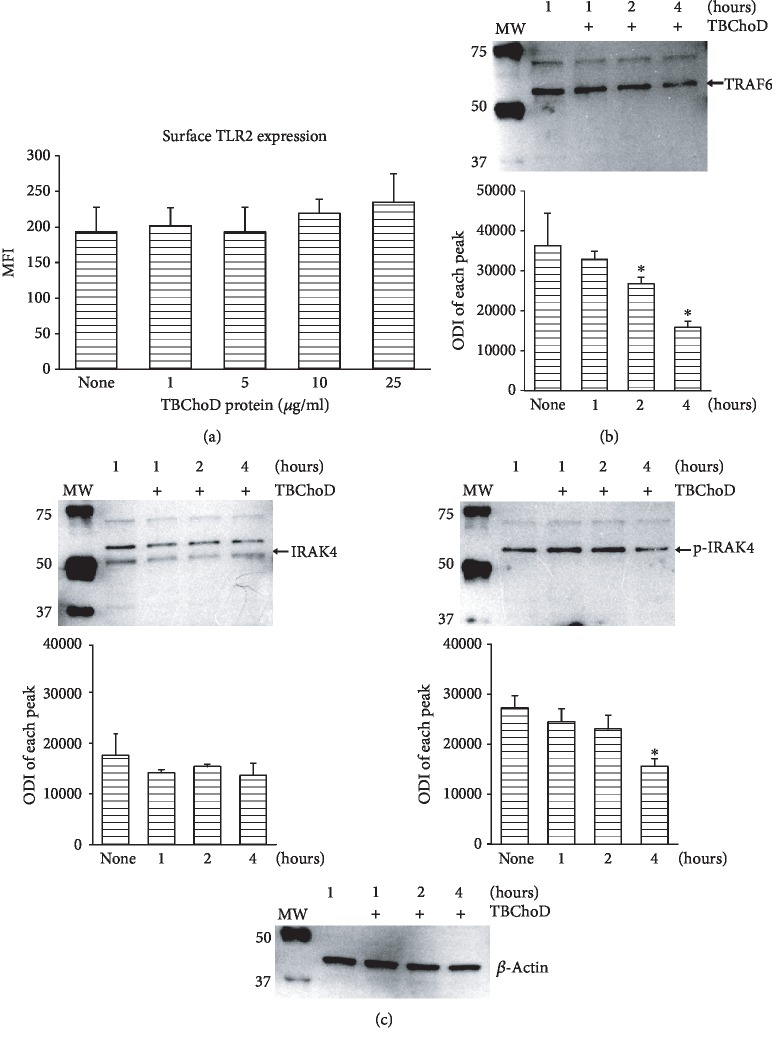
The effect of TBChoD recombinant protein on TLR2, TRAF6, IRAK4, and p-IRAK4 levels in macrophages. (a) The macrophages were treated with a range concentration of TBChoD recombinant protein (1-25 *μ*g/ml) for 24 hours. The cells were then stained with the PE-conjugated anti-TLR2 mAb or a specific isotype control for 30 minutes. The TLR2 expression on the macrophages was analysed using flow cytometry. The graphs show the mean values of the mean fluorescence intensity (MFI) ± SEM from 5 independent experiments. (b, c) The macrophages were treated with 10 *μ*g/ml of TBChoD recombinant protein for 1, 2, or 4 hours. The TRAF6, IRAK4, and p-IRAK4 protein levels were assessed using the immunoblot-ECL method. Representative immunoblots of abovementioned proteins are shown. The bands were quantified by densitometric analysis. The data are presented as the optical density intensity of the area under each band′s peak (ODI) ± SEM from 3 independent experiments. ^∗^Macrophages treated with TBChoD protein vs. nontreated macrophages, *p* ≤ 0.05 (Wilcoxon's signed rank test).

In the next step, the influence of the TBChoD recombinant protein on the TLR2-related signalling proteins was determined. As it is demonstrated in Figure 7(a), this protein had no effect on the TLR2 expression until 24 hours of treatment, independently of the concentration used. To confirm that the drop in the level of IRAK4 and TRAF6 observed by us in macrophages infected with wild-type Mtb, but not mutant strain, is related to cholesterol oxidase, the influence of the TBChoD recombinant protein on the level of both signalling proteins was determined. Our studies demonstrate that two or four hours of treatment significantly lowered the cytosolic level of TRAF6 signalling protein in macrophages (Figure 7(b)). Although, at the same time IRAK4 level was unchanged, its phosphorylation status decreased after 4 hours of incubation with TBChoD (Figure 7(c)).

### 3.4. IL-8 and IL-10 Secretion and Gene Expression in Mtb-Infected Macrophages

**Figure 8:**
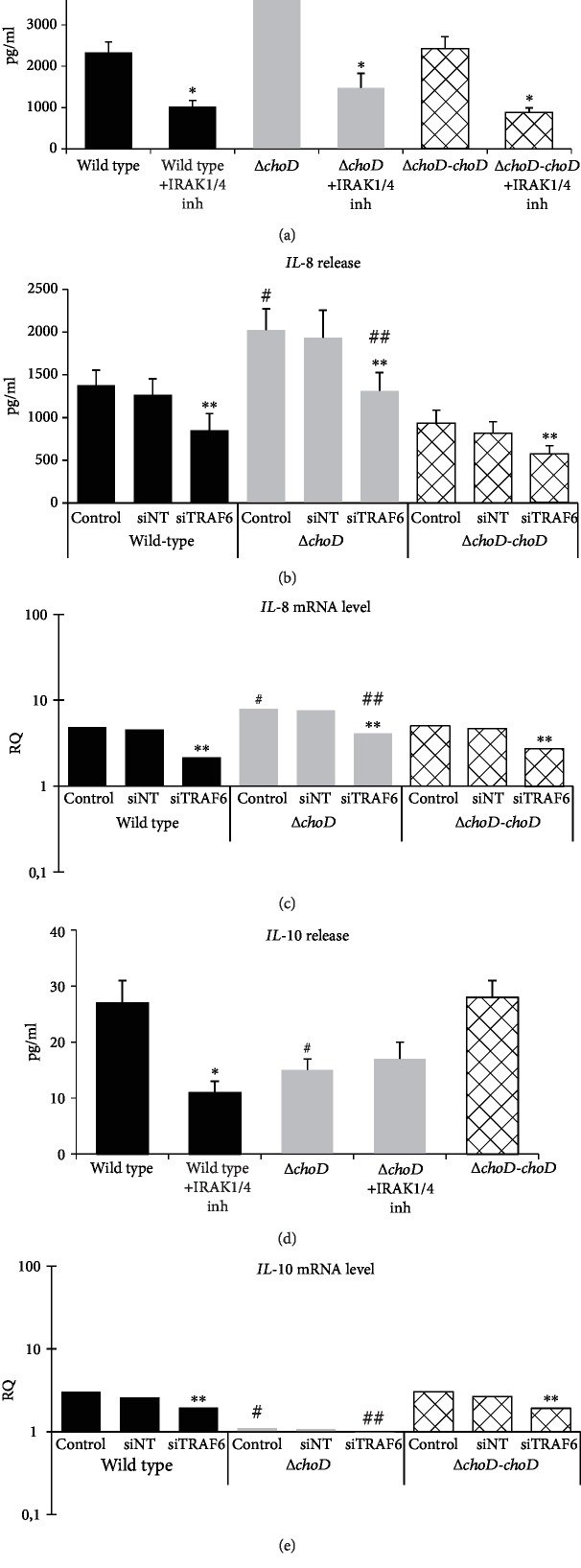
IL-8 and IL-10 secretion and gene expression in Mtb-infected macrophages. (a, d) The macrophages incubated with an IRAK1/4 inhibitor or untreated were infected with the wild-type, Δ*choD*, or Δ*choD-choD* strains for 24 hours. The (a) IL-8 and (d) IL-10 secretion was assessed using a BD CBA kit. The data are presented as pg/ml ± SEM from 5 independent experiments. ^∗^IRAK1/4 inhibitor vs. without IRAK1/4 inhibitor, *p* ≤ 0.01; ^#^Δ*choD* vs. wild-type or Δ*choD-choD*, *p* ≤ 0.05 (all Mann-Whitney *U* test). (b) The macrophages treated with siRNA (either nontargeting or *TRAF6* targeting) or untreated were infected with the wild-type, Δ*choD*, or Δ*choD-choD* Mtb strains for 24 hours. The IL-8 secretion was assessed using a BD CBA kit. The data are presented as pg/ml ± SEM from 5 independent experiments. ^∗∗^siTRAF6 vs. the control, *p* ≤ 0.05; ^#^Δ*choD* vs. wild type or Δ*choD-choD*, *p* ≤ 0.05; ^##^Δ*choD* siTRAF6 vs. wild-type siTRAF6 or Δ*choD-choD* siTRAF6, *p* ≤ 0.05 (all Mann-Whitney *U* test). (c, e) Macrophages treated with siRNA (either nontargeting or *TRAF6* targeting) or untreated were infected with the wild-type, Δ*choD*, or Δ*choD-choD* Mtb strains for 24 hours. The (c) *IL-8* and (e) *IL-10* mRNA levels were measured using qRT-PCR. The data are presented as the mean relative quantification (RQ); *n* = 5. RQ represents the fold change in the gene expression in the infected macrophages compared to the noninfected macrophages, calculated using the ABI 7900HT (RQ) Manager Software (v1.2) and DataAssist Software v3.01 (Thermo Fisher Scientific). ^∗∗^siTRAF6 vs. the control, *p* ≤ 0.05; ^#^Δ*choD* vs. wild type or Δ*choD-choD*, *p* ≤ 0.05; ^##^Δ*choD* siTRAF6 vs. wild-type siTRAF6 or Δ*choD-choD* siTRAF6, *p* ≤ 0.05 (all *t*-test). Control: Mtb-infected macrophages untreated with siRNA; siNT: Mtb-infected macrophages treated with nontargeting siRNA; siTRAF6: Mtb-infected macrophages with a silenced *TRAF6* gene.

The level of IL-8 released by the noninfected macrophages was very low (57 ± 10 pg/ml). We observed that the Mtb lacking a functional copy of the *choD* gene stimulated phagocytes to produce significantly more IL-8 than did wild-type Mtb (4246 pg/ml versus 2329 pg/ml, respectively). Additionally, the complemented strain (Δ*choD-choD*) caused IL-8 levels to reach 2429 pg/ml, which is similar to wild-type Mtb (Figure 8(a)). Moreover, macrophage infection with the Δ*choD* Mtb resulted in a significantly greater mRNA IL-8 level than did infection with wild-type Mtb (RQ = 7963 versus RQ = 4844, respectively) (Figure 8(c)). To assess whether IRAK4 and TRAF6 are functional targets for Mtb to the induction of IL-8 production, we incubated the macrophages with an IRAK1/4 inhibitor prior to the infection. The results showed a similar inhibition level of IL-8 secretion by the phagocytes infected with the wild-type, Δ*choD*, or complemented strain by 56%, 65%, and 63%, respectively (Figure 8(a)). Notably, the IL-8 level from the noninfected phagocytes in the presence of the IRAK1/4 inhibitor was 45 ± 4 pg/ml. Moreover, silencing the *TRAF6* gene in the macrophages led to similar results. The macrophages treated with siTRAF6 secreted significantly less IL-8 than did control macrophages without silenced *TRAF6* in response to infection with the wild-type, Δ*choD*, or complemented strain (by 62%, 65%, and 61%, respectively) (Figure 8(b)). Comparable results were observed in the IL-8 mRNA level. Macrophages with a silenced *TRAF6* gene, infected with each of the Mtb strains, demonstrated significantly lower *IL-8* expression (Figure 8(c)).

In contrast to the proinflammatory IL-8, the immunosuppressant IL-10 was secreted in greater amounts by macrophages in response to wild-type Mtb and the complemented strain than to the Δ*choD* strain (27 pg/ml, 28 pg/ml, and 15 pg/ml, respectively). Noninfected cells produced a very low amount of IL-10 (1.8 ± 0.6 pg/ml) (Figure 8(a)). These data were confirmed by evaluating the *IL-10* gene expression (mRNA), which was significantly greater in the macrophages infected with the wild-type (RQ = 3056) and complemented strain (RQ = 3012) than in noninfected macrophages but not in macrophages infected with the mutant lacking cholesterol oxidase (RQ = 1095) (Figure 8(d)). The presence of an IRAK1/4 inhibitor decreased IL-10 secretion by wild-type-infected phagocytes (by approximately 60%) but did not influence the level of IL-10 secreted by macrophages infected with the mutant lacking the gene encoding for cholesterol oxidase. The complemented strain affected IL-10 secretion in a similar manner as the wild type (Figure 8(d)). When the *TRAF6* gene was silenced in the macrophages, we observed that the *IL-10* mRNA level in response to the wild-type Mtb infection was significantly lower compared to the control phagocytes (also infected but without *TRAF6* silencing). Compared with the control, silencing *TRAF6* in mutant macrophages lacking cholesterol oxidase did not change the *IL-10* mRNA level (Figure 8(e)).

Additionally, the THP-1 transcription efficiency was assessed for both the TRAF6 mRNA and protein level. The results indicate that *TRAF6* gene expression was lower in transfected cells than in control cells, which was reflected by a decrease in the TRAF6 protein level (Suppl. [Supplementary-material supplementary-material-1]).

We also tested the secretion of IL-1*β*, IL-6, and TNF-*α* by noninfected and infected macrophages. However, there was no difference between wild-type Mtb and mutant strain to the induction of macrophages to release all the abovementioned cytokines (Table 1). Therefore, they were excluded from other analysis.

## 4. Discussion

Although TLR2 signalling is a suggested mechanism by which Mtb affects the macrophages' effector functions to avoid being killed and to modulate the adaptive immune response, the interaction of tubercle bacilli with the TLR2-mediated pathway is not fully investigated and understood [15, 39]. The engagement of TLR2 by Mtb antigens or the whole bacterial cell stimulates macrophages to enhance the immunosuppressive IL-10 production and decrease IL-12 production, resulting in diminished protective responses of T helper type 1 cells [40, 41]. Moreover, IL-10 is known to block phagosome maturation and consequently lower the bactericidal activity of phagocytes [42]. The downregulation of TLR2 and MyD88 by Mtb is essential for its escape from the phagolysosome to the cytosol [43]. The TLR2-MyD88 pathway is also used by tubercle bacilli to induce apoptosis of the infected macrophages [44]. Moreover, our previously published data [32] clearly indicate that blocking the IRAK1 and 4 proteins decreased the ability of Mtb to inhibit the reactive oxygen species generation by PMA-stimulated macrophages. Furthermore, the determinative role of TLR2 in the Mtb infection was supported by Tjärnlund et al. [45] who demonstrated a greater susceptibility of TLR2^−/−^ mice to Mtb infection compared to wild-type mice. Thus, TLR2-mediated signalling is an intriguing pathway to study, one that may help better recognize and understand the intracellular survival capabilities of Mtb.

Several mycobacterial products including LAM, manLAM, 19 kDa protein, and PIM are the best known TLR2 agonists that also serve as Mtb virulence factors [3, 8]. However, new potential targets for TLR2 that modulate the macrophages' function are also being revealed. Those worth mentioning include cell envelope-associated serine hydrolase [46] and the PEE family proteins [47]. Our group is studying the correlation between Mtb virulence and the bacteria's ability to produce enzymes participating in cholesterol degradation [32–34]. Notably, our previously published data clearly show that ChoD contributes to Mtb virulence and lowers the bactericidal activity of human macrophages [32].

The purpose of the studies presented here was to determine the impact of wild-type Mtb H37Rv and its mutant unable to produce cholesterol oxidase as well as the TBChoD recombinant protein on the expression of TLR2 and the activation of its signalling proteins as an important mediator of the phagocyte proinflammatory response to Mtb infection. To the best of our knowledge, the current study is the first to assess the molecular mechanism of the ChoD effect as a factor disturbing the TLR2-mediated signalling pathway in human macrophages.

We found that none of the tested strains influenced the macrophages' surface expression and mRNA level of TLR2, regardless of the infection time (0.5–24 hours). Similarly, TBChoD recombinant protein also did not influence the surface expression of TLR2. These results indicate that Mtb recognition by TLR2 does not affect this receptor's expression on macrophages regardless of the presence or absence of ChoD. However, in a separate study using a mouse macrophage model, Mtb H37Rv downregulated the *TLR2* gene expression after 48 hours of infection [43].

In the current study, we reported that wild-type Mtb, but not the ∆*choD* strain, decreased the cytosolic IRAK4 and TRAF6 levels after prolonged (24 hours) infection. Blocking TLR2 with an anti-TLR2 mAb effectively inhibited the influence of the wild-type Mtb on the abovementioned signalling proteins which suggest the importance of the surface expression of this receptor in Mtb action. An important consequence of the decreased cytosolic TRAF6 level in Mtb-infected macrophages can be the inhibition of the downstream MAPK activity [12]. Our previous study demonstrates that wild-type Mtb blocks the ability of phorbol ester to induce the phosphorylation of ERK1/2 (which is involved in the MAPK pathway) in macrophages. However, both strains caused no decrease in the cytosolic level of ERK1/2 [32]. Since TLR2 signalling plays a key role in the macrophages' proinflammatory effect and in the bactericidal and Th1-mediated immune response [9–13], the ability of Mtb to disrupt this pathway can facilitate the bacteria to modulate immune functions and thus survive [32]. However, the mycobacterial ligands that elicit a modulatory effect of the TLR2 signalling pathway are not well characterized. Pathak et al. [48] proposed that ESAT-6 in the Mtb is responsible for attenuating IRAK4 interaction with MyD88 in mouse macrophages. In the current study, we demonstrated that the Mtb mutant lacking ChoD (∆*choD*) had no effect on the IRAK4 and TRAF6 signalling proteins in human macrophages, regardless of the infection time. However, what is important and needs to be underlined is that the TBChoD recombinant protein decreased the cytosolic level of TRAF6 already after 2 hours of treatment and strongly lowered the phosphorylation of IRAK4. It is much easier for macrophages to respond to the nonbound recombinant protein than to ChoD associated with bacteria; therefore, the targeting of IRAK4 and TRAF6 is faster. Our results clearly indicate that cholesterol oxidase is required for tubercle bacilli to generate a negative signal into the macrophages and decrease the activation of the TLR2 signalling pathway. As we demonstrated previously [32], only the ∆*choD* strain induced the bactericidal activity of the macrophages. Using an IRAK1/4 inhibitor or blocking TLR2 *via* an anti-TLR2 mAb caused the mutant strain lacking *choD* to behave similar to wild-type Mtb. Therefore, our present and former studies strongly suggest that Mtb employs ChoD to generate a negative signal on the TLR2-dependent signalling pathway.

We found here that both of the tested strains significantly enhanced the level of *IRAK4* mRNA, with wild-type Mtb stimulating the greatest increase. Moreover, only the wild-type strain increased the *TRAF6* mRNA level in a statistically significant manner. In contrast, Rahman et al. [43] demonstrated that Mtb H37Rv downregulated the *IRAK4* and *TRAF6* gene expression in peritoneal mouse macrophages after a prolonged infection of 48 hours. The observed differences between our results and those of Rahman et al. [43] can be related to the different time chosen to study the IRAK4 and TRAF6 mRNA levels. It is accepted that Mtb induces time-dependent up- or downregulation of various genes' transcription in macrophages [49, 50]. Therefore, during a short infection time, the *IRAK4* and *TRAF6* mRNA levels can be unaffected, then upregulated (24 hours), and finally downregulated after a prolonged infection time (48 hours). The decreased IRAK4 and TRAF6 protein levels observed here with simultaneously enhanced mRNA levels in the macrophages infected with the Mtb wild type seem to look discrepant. However, ingested tubercle bacilli induced various processes in macrophages, e.g., time-dependent up- or downregulation of genes' transcription [49, 50]. Hence, we can hypothesize that Mtb exerts a time-dependent, sometimes opposite effect on macrophages' biological activity to survive, and the Mtb-macrophage interaction is dynamic. The observed downregulation of the IRAK4 and TRAF6 proteins in the cytosol is probably due to a regulatory negative feedback. Mtb primarily overactivates the TLR2 pathway and eventually causes its silencing, which leads to the unresponsiveness of macrophages to infection. One study suggests that prolonged TLR2 signalling activation might allow the Mtb to persist as a chronic infection [39]. To verify this hypothesis, we stimulated macrophages with LTA, hrIL-1*β*, and IL-1R/TLR2 activators [51, 52] for 24 hours. We observed a negative signal that induced IRAK4 and TRAF6 degradation. Extensive and/or prolonged TLR stimulation with various ligands results in decreased cytosolic IRAK1, IRAK4, and TRAF6 and eventually induces self-tolerance [37, 38, 53]. Extensive stimulation of innate immune cells (including macrophages and neutrophils) *via* bacterial antigens such as lipopolysaccharide can stimulate harmful inflammation. Therefore, cells' tolerance to bacterial stimuli is an adaptive mechanism protecting the host cell against damage [54]. However, based on our studies, we can propose a model in which TLR2-dependent macrophages' tolerance can also be used by the Mtb to limit the proinflammatory cytokine production and reactive oxygen species formation, allowing the bacteria to survive. Notably, a similar negative regulation of TLR signalling is also observed in some viral infections. NS3/4A, a serine protease of the hepatitis C virus, caused TRIF proteolysis, an adaptor protein of TLR3, resulting in limiting the host defence gene expression and the persistence of the pathogen [55].

The infection of macrophages with Mtb leads to the production and secretion of various cytokines, chemokines, and other antibacterial agents [10, 56]. Wild-type and ∆*choD* Mtb substantially differed in how they stimulated macrophages to produce the chemotactic cytokine IL-8 and the immunosuppressive cytokine IL-10. It should be noted that although wild-type Mtb decreased the cytosolic level of IRAK4 and TRAF6, it also successfully activated macrophages to cytokine production. We hypothesize that wild-type Mtb initially activate macrophages (almost up to 4 hours) through TLR2, which is sufficient to induce cytokine production.

Depriving the tubercle bacilli of the gene encoding for cholesterol oxidase resulted in Mtb inducing less of an immunosuppressive response of the phagocytes. To prove that the TLR2-mediated signalling pathway is used by Mtb to disrupt macrophages' functional response, we blocked the activity of the TLR2-related signalling proteins in macrophages before infection. Notably, both the pharmacological inhibitor of IRAK1/4 and siRNA targeting TRAF6 significantly abrogated the ability of both Mtb strains to stimulate *IL-8* expression. It can indicate a significant involvement of the TLR2-dependent pathway in IL-8 production, stimulated by Mtb. However, this effect occurred independent of the presence or absence of cholesterol oxidase in the tubercle bacilli. In contrast, the production of IL-10 by phagocytes treated with the IRAK1/4 inhibitor or siRNA targeting TRAF6 was reduced, but only in the cells infected with the wild-type strain. These results indicate that the TLR2-mediated signalling pathway is necessary to induce the immunosuppressive effect of the wild-type Mtb [14]. Moreover, it was ChoD of Mtb that is responsible for immunosuppressive response of macrophages. Lower IL-10 production initiated by mutant strain can support this hypothesis.

IL-10 is known to block phagosome maturation, which facilitates Mtb intracellular survival [57]. As it was previously clearly described by us [32], the wild-type Mtb replicates inside macrophages significantly better than the Mtb mutant lacking ChoD.

## 5. Conclusion

In summary, TLR2 signalling has the capacity to modulate macrophages' response to Mtb infection. However, the engagement of TLR2 with wild-type Mtb or with cholesterol oxidase itself, generates rather negative signals to macrophages. What is more, cholesterol oxidase is a virulence factor of tubercle bacilli that enables Mtb to affect the TLR2 signalling pathway to manipulate the macrophages' response. These studies give a new proposition to a better understanding of the immune evasion of Mtb.

## Figures and Tables

**Table 1:** Secretion of cytokines by Mtb-infected macrophages. The macrophages were infected with the wild-type or Δ*choD* strains, or remained uninfected (none) for 24 hours. The secretion of cytokines was assessed using a BD CBA kit. The data are presented as pg/ml ± SEM from 5 independent experiments.

	None	Wild-type Mtb	Δ*choD*
IL-1*β*	13 ± 2	61 ± 14	71 ± 5
IL-6	1.3 ± 0.1	93 ± 68	107 ± 63
TNF-*α*	8 ± 10	748 ± 181	530 ± 276

## Data Availability

The data used to support the findings of this study are included within the article.

## Supplementary Materials

Supplementary Materials Suppl. Figure 1: analysis of the obtained TBChoD recombinant protein. Suppl. Figure 2: verification of the TRAF6 gene silencing efficiency in THP-1 cells.
