# Supplementary material for: Mycobacterium tuberculosis Requires Cholesterol Oxidase to Disrupt TLR2 Signalling in Human Macrophages
Source: Mediators Inflamm. 2019 Dec 1;2019:2373791. doi: 10.1155/2019/2373791 (PMC6913169; doi:10.1155/2019/2373791)
Supplement: Supplementary Materials — Suppl. Figure 1: analysis of the obtained TBChoD recombinant protein. Suppl. Figure 2: verification of the TRAF6 gene silencing efficiency in THP-1 cells. [file 2373791.f1.pdf]

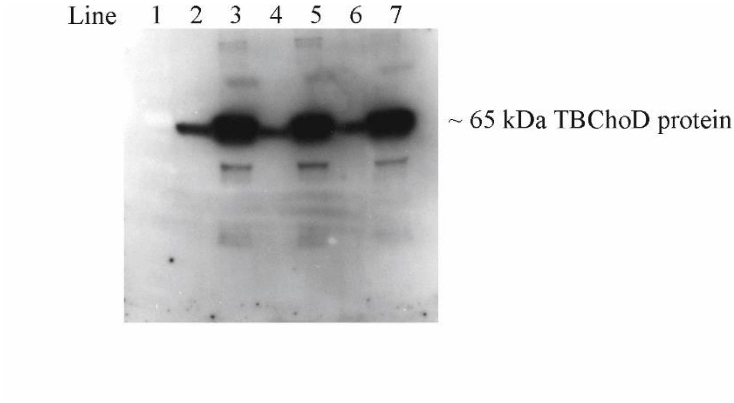

Suppl. Fig. 1

Suppl. Figure 1. Analysis of obtained TBChoD recombinant protein.
Western blot analysis of *M. smegmatis* lysates overproducing of TBChoD protein. Lanes: 1 - PageRuler^{TM} Plus Prestained Protein Ladder; 2, 4, 6 – *M. smegmatis* with TBchoDJam-6xHis without acetamide induction; 3, 5, 7 – *M. smegmatis* with TBchoDJam-6xHis with acetamide induction.

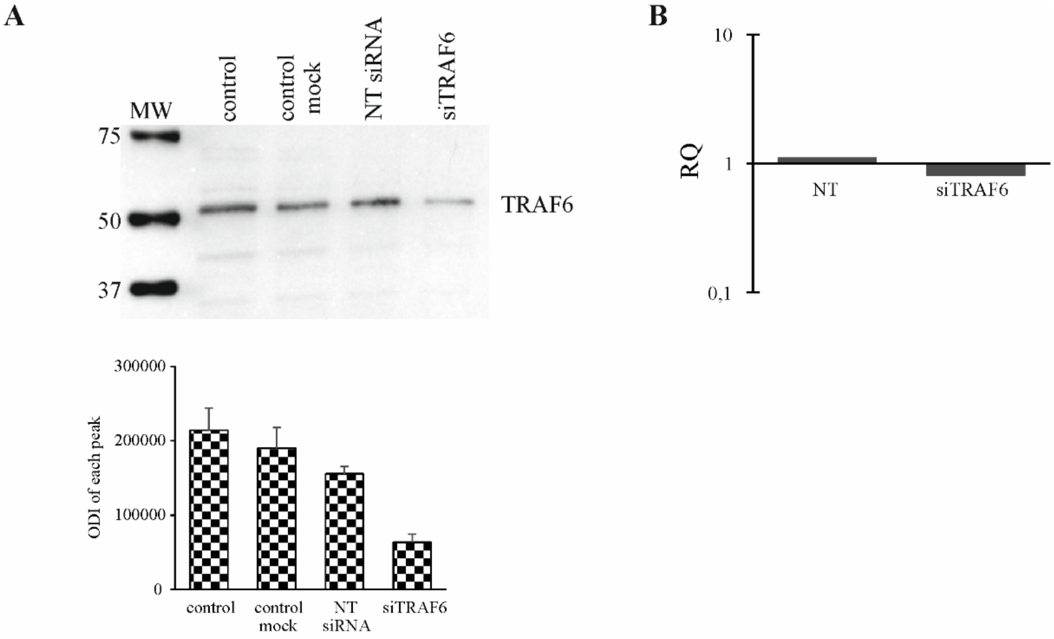

Suppl. Fig. 2

Suppl. Figure 2. Verification of the *TRAF6* gene silencing efficiency in THP-1 cells.

Macrophages were treated with siRNA (either non-targeting or TRAF6 targeting) or untreated and incubated for 24 hours. Next, the cells were lysed, and total RNA was isolated. **(A)** The TRAF6 protein level was assessed using the immunoblot-ECL method. A representative immunoblot is shown. The bands were quantified by densitometric analysis. The data are presented as the optical density intensity of the area under each band's peak (ODI) ± SEM from 3 independent experiments. Control – macrophages not treated with siRNA; control mock - macrophages treated with the transfection agent without siRNA; NT siRNA - macrophages treated with non-targeting siRNA; siTRAF6 - Mtb-infected macrophages with a silenced *TRAF6* gene. **(B)** The *TRAF6* mRNA level was measured using qRT-PCR. Data are presented as the mean relative quantification (RQ) from 3 independent experiments. The RQ represents the fold change in the gene expression in the infected macrophages compared to the non-infected macrophages, calculated using ABI 7900-HT (RQ) manager software (v1.2) and DataAssist software v3.01 (Thermo Fisher Scientific).
